# Investigating potential molecular mechanisms linking 6PPD-quinone exposure to Intervertebral Disc Degeneration: A network toxicology, molecular docking, and molecular dynamics simulation study

**DOI:** 10.1371/journal.pone.0359621

**Published:** 2026-10-22

**Authors:** Bin Yang, Xiangjin Wang, Li Zhuo, Yutong Chen, Qiansong He

**Affiliations:** 1 Bayi Orthopedic Hospital, China RongTong Medical Healthcare Group Co., Ltd., Chengdu, China; 2 Department of Orthopedics, Hospital of Chengdu University of Traditional Chinese Medicine, Chengdu, China; 3 School of Sports Medicine and Health, Chengdu Sports University, Chengdu, China; Manipal Academy of Higher Education, INDIA

## Abstract

As an emerging pollutant derived from tire rubber, 6PPD-quinone (6PPD-Q) has raised public health concerns due to widespread environmental exposure; however, its potential toxicological effects on avascular tissues such as intervertebral discs remain unclear. This study integrated network toxicology, molecular docking, and molecular dynamics (MD) simulation to investigate the potential mechanisms linking 6PPD-Q exposure to intervertebral disc degeneration (IVDD). ADMET toxicological predictions indicated high model-predicted probabilities for SR-MMP and SR-ARE activation. Through multidimensional database integration, 59 common targets potentially associated with 6PPD-Q and IVDD were identified. Protein–protein interaction network analysis further identified five hub genes: PTGS2, MMP2, SMAD3, RELA, and GSK3B. Functional enrichment analysis suggested that these targets were mainly involved in AGE-RAGE, PI3K-Akt, MAPK, TNF, and IL-17 signaling pathways, collectively implicating oxidative stress, inflammation, and apoptosis. Molecular docking showed favorable predicted binding between 6PPD-Q and the five hub proteins, with RELA exhibiting the most favorable docking score (−8.82 kcal/mol). A subsequent 100-ns MD simulation of the 6PPD-Q–RELA complex showed relatively stable RMSD, Rg, and SASA profiles and a concentrated low-energy conformational basin, supporting its dynamic stability under the simulated conditions. Collectively, these computational findings suggest potential molecular links between 6PPD-Q exposure and IVDD and provide hypotheses for subsequent experimental validation.

## Introduction

Intervertebral disc degeneration (IVDD) is the primary pathological basis of low back pain (LBP) [1]. Epidemiological data from 2019 indicate that there are over 568 million people worldwide suffering from LBP, making it the leading cause of disability and imposing a heavy healthcare burden [2]. The intervertebral disc (IVD) consists of the nucleus pulposus (NP), the annulus fibrosus, and the cartilaginous endplates, and is responsible for supporting the spine and distributing stress [3]. The core pathological features of IVDD are decreased NP cell activity and an imbalance in extracellular matrix (ECM) degradation [1,4]. Current evidence suggests that a vicious cycle of mitochondrial dysfunction and oxidative stress (OS) disrupts the homeostasis of the NP microenvironment and accelerates the degenerative process [5,6]. Therefore, identifying potential environmental risk factors that disrupt IVD mitochondrial homeostasis is of great significance for the prevention and intervention of IVDD.

N-(1,3-dimethylbutyl)-N’-phenyl-p-phenylenediamine (6PPD) is a commonly used antioxidant in tire rubber. Upon exposure to air, it readily reacts with ozone to form 6PPD-quinone (6PPD-Q), a highly polar and water-soluble compound [7–9]. As tires wear down, 6PPD-Q is widely released into surface runoff, soil, and PM2.5 [10,11]. This environmental distribution leads to inevitable long-term human exposure through inhalation, ingestion, and skin contact [12]. Recent biomonitoring studies have frequently detected 6PPD-Q not only in the urine and blood of the general population but even in the placentas of pregnant women [13,14], suggesting that this compound poses a real threat to human health.

Currently, molecular toxicological studies on 6PPD-Q are beginning to emerge. In vitro and in vivo studies indicate that exposure to 6PPD-Q not only causes multi-organ dysfunction, including damage to the nervous, hepatic, and respiratory systems, but also exhibits significant cytotoxicity toward various human cell lines (e.g., HepG2 and L02) [15–18]. Its core toxicological mechanisms primarily involve disrupting redox homeostasis, inhibiting endogenous antioxidant systems, and inducing DNA damage [11,18]. Crucially, multiple in vitro studies have clearly demonstrated that 6PPD-Q exposure abnormally upregulates cellular mitochondrial oxygen consumption, profoundly disrupting cellular energy metabolism homeostasis [19]. Therefore, mitochondrial dysfunction and OS may represent key nodes in the 6PPD-Q toxicity pathway.

Although systemic toxicity of 6PPD-Q has been reported, its effects on the musculoskeletal system, particularly IVDs, remain unclear. Adult IVDs are the largest avascular organs in the human body, and their nutritional metabolism is highly dependent on diffusion through the cartilaginous endplates [20]. This physiological characteristic makes them susceptible to the local diffusion and accumulation of environmental toxins from the bloodstream [21]. Concurrently, NP cells, situated in a microenvironment characterized by hypoxia and high mechanical stress, are extremely sensitive to oxidative damage [22]. Given the toxicological profile of 6PPD-Q, which induces mitochondrial damage and OS, environmental exposure to 6PPD-Q may disrupt the homeostasis of the IVD microenvironment and drive the progression of intervertebral disc disease. However, the specific molecular targets and signaling networks involved remain to be elucidated.

Combining computational toxicology with molecular docking and molecular dynamics simulation provides an effective computational approach for evaluating the potential molecular interactions between environmental chemicals and disease-related targets [23]. This study employs the aforementioned methods to evaluate the potential impact of 6PPD-Q exposure on IVDD. By integrating public databases to identify shared targets, constructing protein-protein interaction (PPI) networks, and performing pathway enrichment analysis, we systematically mapped the potential molecular network linking 6PPD-Q exposure to IVDD. Subsequently, molecular docking was used to evaluate the potential binding affinity of 6PPD-Q with key targets. The protein–ligand complex with the most favorable docking score was further subjected to molecular dynamics simulation to assess its conformational behavior and dynamic stability. This study aims to provide computational toxicological evidence for the potential association between 6PPD-Q exposure and IVDD and to offer target references for subsequent experimental validation.

## Methods

### Prediction of the toxicological profile of 6PPD-Q

The chemical structure, molecular formula, and Simplified Molecular Input Line Entry System (SMILES) string of 6PPD-Q were obtained from the PubChem database (https://pubchem.ncbi.nlm.nih.gov/) [24]. Subsequently, the SMILES string was imported into the ADMETLAB 3.0 platform (https://admetlab3.scbdd.com/) to predict and evaluate the toxicological characteristics of the compound.

### Screening and standardization of potential targets for 6PPD-Q

We utilized four online databases—ChEMBL [25], SEA [26], TargetNet [27], and Swiss Target Prediction [28]—in combination to identify potential targets for 6PPD-Q, strictly limiting the search to the species “Homo sapiens.” After merging the search results and removing duplicates, all identified targets were entered into the UniProt database (https://www.uniprot.org/) [29] for standardization, thereby constructing a standardized target library for 6PPD-Q.

### Acquisition and intersection analysis of IVDD disease targets

Using “Intervertebral disc degeneration” as the search term, we collected data related to IVDD from the GeneCards database (www.genecards.org) [30], the OMIM database (https://www.omim.org/) [31], and Ensembl (https://www.ensembl.org) [32] to collect disease-associated genes related to IVDD. After merging the above data and removing duplicates, an IVDD disease target set was established. Subsequently, Venn diagram analysis was used to map the 6PPD-Q target library against the IVDD target set, and the genes in their intersection were extracted as common intervention targets for subsequent studies.

### Construction of protein-protein interaction (PPI) networks

The intersected targets were imported into the STRING database (https://string-db.org/) [33] to assess protein-protein interactions, with the species set to “Homo sapiens” and the confidence score threshold set to > 0.4. After extracting the interaction data, Cytoscape 3.10.1 software was used to construct and visualize the PPI network. Furthermore, the built-in CentiScaPe 2.2 plugin was employed to calculate the topological characteristics of network nodes (such as degree centrality and betweenness centrality), and core targets were identified within the network based on these metrics.

**Gene Ontology (GO) and Kyoto Encyclopedia of Genes and Genomes (KEGG) enrichment analysis**The Metascape platform (https://metascape.org) [34] was used to perform GO and KEGG pathway enrichment analyses on the intersection of target genes. The GO analysis system covers three dimensions: Biological Processes (BP), Cellular Components (CC), and Molecular Functions (MF). For pathway and process enrichment analysis, the minimum overlap was set to 3, the raw P-value cutoff was set to 0.01, and the minimum enrichment factor was set to 1.5. Enrichment P-values were calculated using the hypergeometric test, and Benjamini–Hochberg correction was applied to account for multiple hypothesis testing. Terms with an adjusted q-value < 0.05 were considered statistically significant. Enriched terms meeting these criteria were ranked according to statistical significance, and the top 10 GO terms and top 20 KEGG pathways were selected for visualization. To further visualize the relationships between hub genes and significantly enriched signaling pathways, a target–pathway interaction network was constructed using Cytoscape based on the identified core targets and representative KEGG pathways.

### Molecular docking

To validate the binding affinity between 6PPD-Q (ligand) and the core target (receptor), molecular docking simulations were performed in this study. First, the 3D structure of 6PPD-Q was downloaded from the PubChem database and converted to Mol2 format using OpenBabel 3.1.1; it was then imported into AutoDock Tools 1.5.7 to perform hydrogenation, calculate charges, and export it as a PDBQT ligand file. For the receptor protein, the high-resolution 3D crystal structure of the core target was retrieved and downloaded from the RCSB PDB database. Using PyMOL 3.6.1, remove water molecules and heteroatoms from the original structure, then convert it to PDBQT format using AutoDock Tools. Molecular docking was performed using AutoDock 4 with the receptor treated as rigid and the ligand treated as flexible. For each receptor–ligand pair, the conformation with the lowest predicted binding energy was selected as the representative docking pose for subsequent visualization and interaction analysis. The optimal docking conformations were visualized using PyMOL.

### Molecular dynamics simulation

The protein–ligand complex with the most favorable docking score was selected for subsequent molecular dynamics (MD) simulation to evaluate its dynamic stability. MD simulations were performed using GROMACS 2025.3 [35]. The protein was parameterized using the AMBER14SB force field, while the ligand topology was generated using Sobtop. The protein–ligand complex was placed in a periodic cubic box, solvated using the TIP3P water model, and supplemented with ions to achieve a physiological NaCl concentration of 150 mM. The system was subjected to 500 steps of conjugate-gradient energy minimization with a convergence tolerance of 100.0 kJ/mol·nm and a step size of 0.01 nm, followed by a 100-ps restrained equilibration with a time step of 1 fs. A 100-ns production MD simulation was subsequently performed with a time step of 2 fs. The temperature was maintained at 298.15 K using the V-rescale thermostat with a coupling constant of 0.2 ps, and the pressure was maintained at 1.0 bar using the C-rescale barostat. Bonds involving hydrogen atoms were constrained using the LINCS algorithm. Long-range electrostatic interactions were treated using the particle mesh Ewald (PME) method with a cutoff of 1.0 nm, while van der Waals interactions were handled using the Verlet cutoff scheme with a cutoff distance of 1.0 nm. Dispersion corrections for energy and pressure were applied. Center-of-mass motion was removed during the simulations. Trajectory analyses included root-mean-square deviation (RMSD), root-mean-square fluctuation (RMSF), radius of gyration (Rg), solvent-accessible surface area (SASA), and the number of protein–ligand hydrogen bonds. In addition, two-dimensional and three-dimensional free-energy landscapes (FELs) were constructed using RMSD and Rg as collective variables to characterize the conformational energy distribution of the complex.

## Results

### Assessment of the toxicological profile of 6PPD-Q

The molecular characteristics of 6PPD-Q, including its two-dimensional chemical structure and three-dimensional conformation, are presented in Fig 1. The molecular formula of 6PPD-Q is C18H22N2O2, with a molecular weight of 298.4 g/mol (S1 Table). Prediction model results based on the ADMETLAB 3.0 platform (Table 1) indicate that 6PPD-Q exhibits significant hepatotoxicity (H-HT: 0.830), a risk of drug-induced liver injury (DILI: 0.858), and hematotoxicity (0.740). Its genotoxicity score (0.994) suggests a potential risk of DNA damage associated with this compound. At the mechanistic level, 6PPD-Q exhibits high activation potential for SR-MMP (0.913) and SR-ARE (0.909). These toxicological parameters suggest that the exposure risk of 6PPD-Q involves not only systemic toxicity but may also specifically interfere with OS and matrix degradation processes, which is consistent with a potential association with IVDD-related oxidative stress and matrix homeostasis.

**Fig 1:**
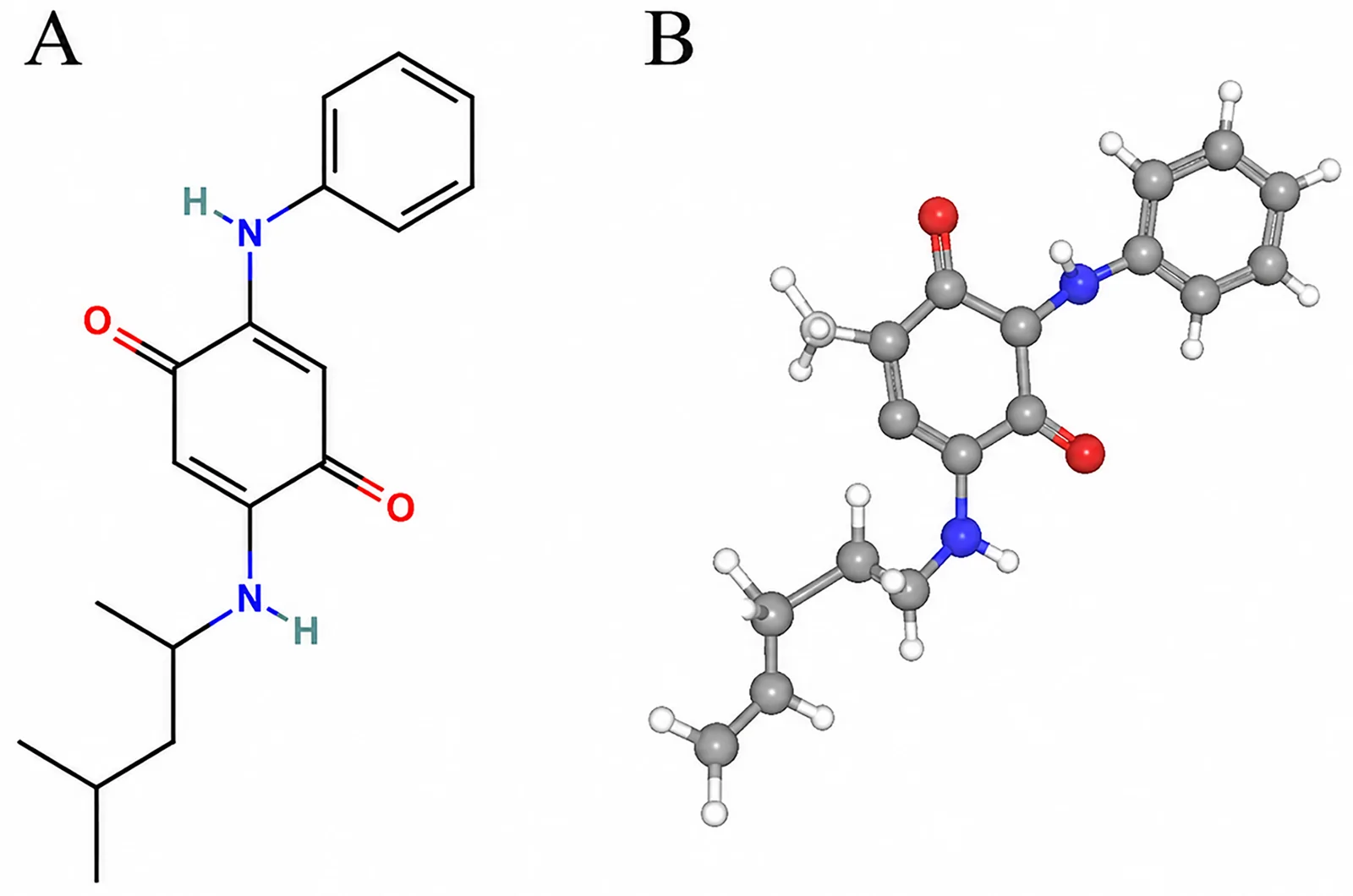
Two-dimensional and three-dimensional structures of 6PPD-quinone. (A) Two-dimensional chemical structure of 6PPD-quinone. (B) Three-dimensional molecular conformation of 6PPD-quinone.

**Table 1:** Prediction of Toxicological Properties for 6PPD-Q Using ADMETLAB.

Classification	6PPD-Q
MW	298.4
BBB	0.032
Carcinogenicity	0.437
DILI	0.858
Genotoxicity	0.994
H-HT	0.830
Hematotoxicity	0.740
hERG	0.257
Nephrotoxicity-DI	0.597
Neurotoxicity-DI	0.570
Ototoxicity	0.221
Respiratory	0.611
SkinSen	0.975
SR-ARE	0.909
SR-MMP	0.913

### Identification of common targets for 6PPD-Q and IVDD

By searching the ChEMBL, SEA, SwissTargetPrediction, and TargetNet databases and removing duplicates, a total of 204 potential binding targets for 6PPD-Q were identified (S2 Table). Concurrently, by integrating the GeneCards, OMIM, and Ensembl databases, 2,236 IVDD-associated genes were identified (S3 Table). Using Venn diagrams to map the aforementioned compound targets against disease targets, 59 common genes were ultimately identified (Fig 2, S4 Table). These overlapping genes constitute the potential molecular basis for 6PPD-Q’s intervention in IVDD.

**Fig 2:**
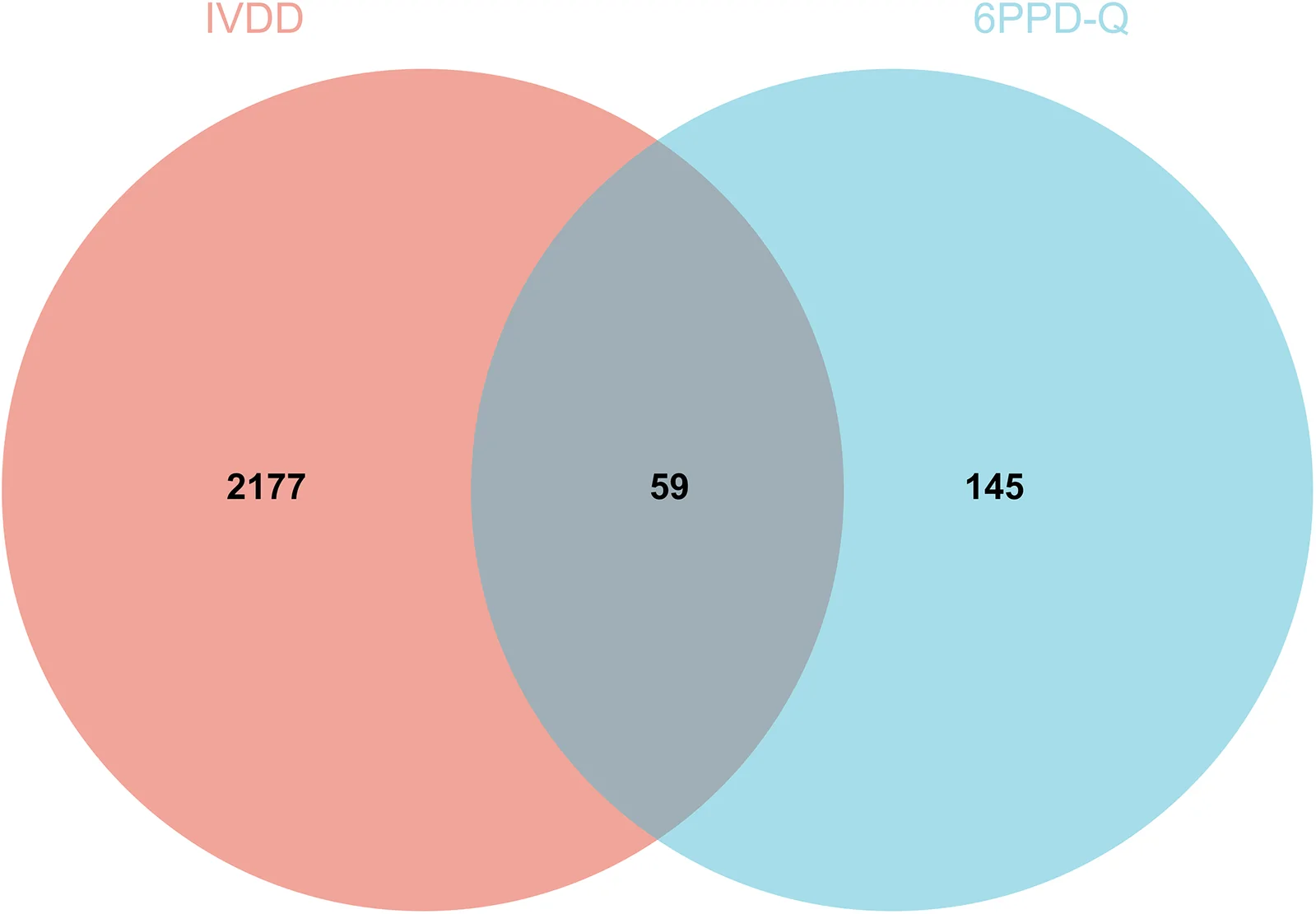
Venn diagram of 6PPD-Q and IVDD-related targets.

### PPI network construction and identification of core targets

To analyze the interactions among the 59 overlapping targets, an initial PPI network comprising 55 nodes and 224 edges (confidence > 0.4) was constructed using the STRING database. Subsequently, network topological parameters were calculated using Cytoscape 3.10.1 and its CentiScaPe 2.2 plugin. Thresholds were set based on the mean values (Degree > 8.145, Betweenness Centrality > 75.745, Closeness Centrality > 0.008), resulting in a core subnetwork comprising 12 nodes and 33 edges. The topological parameters of these 12 core targets are detailed in Table 2. Ranked by Degree, the top five hub genes were PTGS2, MMP2, SMAD3, RELA, and GSK3B (Fig 3).

**Table 2:** Information of 12 core targets.

NO.	Gene Symbol	Gene Name	Degree	Betweenness	Closeness
1	PTGS2	Prostaglandin G/H synthase 2	26	701.215	0.011
2	MMP2	Matrix metalloproteinase-2	21	308.689	0.011
3	SMAD3	Mothers against decapentaplegic homolog 3	20	176.262	0.01
4	RELA	Transcription factor p65	19	178.16	0.01
5	GSK3B	Glycogen synthase kinase-3 beta	19	250.916	0.01
6	MAPK14	Mitogen-activated protein kinase 14	16	118.755	0.01
7	MAP2K1	Dual specificity mitogen-activated protein kinase kinase 1	15	115.071	0.009
8	PDGFRA	Platelet-derived growth factor receptor alpha	14	242.676	0.009
9	ACE	Angiotensin-converting enzyme	11	220.305	0.009
10	NTRK1	High affinity nerve growth factor receptor	9	115.976	0.009
11	CTSK	Cathepsin K	9	135.553	0.008
12	NOS3	Nitric oxide synthase, endothelial	9	115.401	0.009

**Fig 3:**
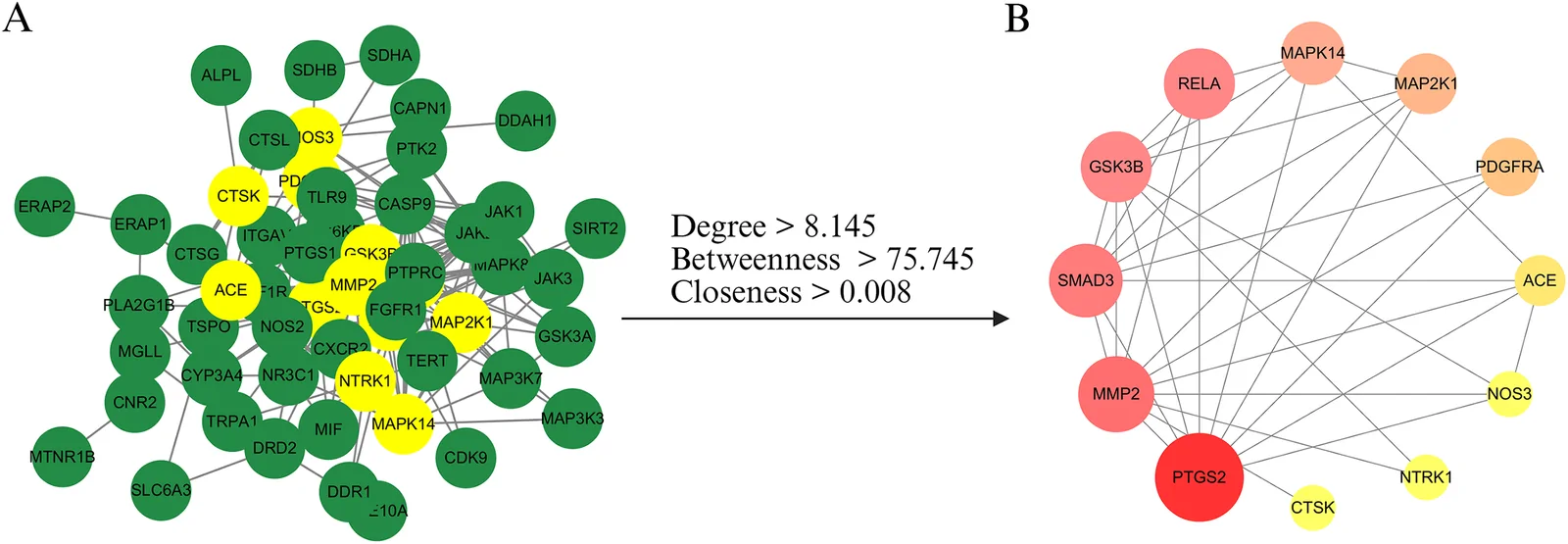
Candidate targets were identified by PPI analysis. A total of 12 key targets were obtained by screening 59 cross targets by DC, BC and CC.

### GO and KEGG pathway enrichment analysis

Enrichment analysis using the Metascape platform further elucidated the biological functions of the aforementioned intersecting targets. The GO analysis identified a total of 797 BP, 58 CC, and 73 MF (Fig 4). Among BP, the targets were significantly enriched in enzyme-linked receptor protein signaling pathways, positive regulation of cell migration, and mitochondrial-related processes such as OS response and apoptosis signaling pathway regulation. In terms of CC, the targets were primarily localized to the outer cell membrane, receptor complexes, and synaptic structures. MF were concentrated in kinase binding, protein kinase activity, and transcription factor binding, which is consistent with the kinase hub genes, such as GSK3B, identified in the PPI network.

**Fig 4:**
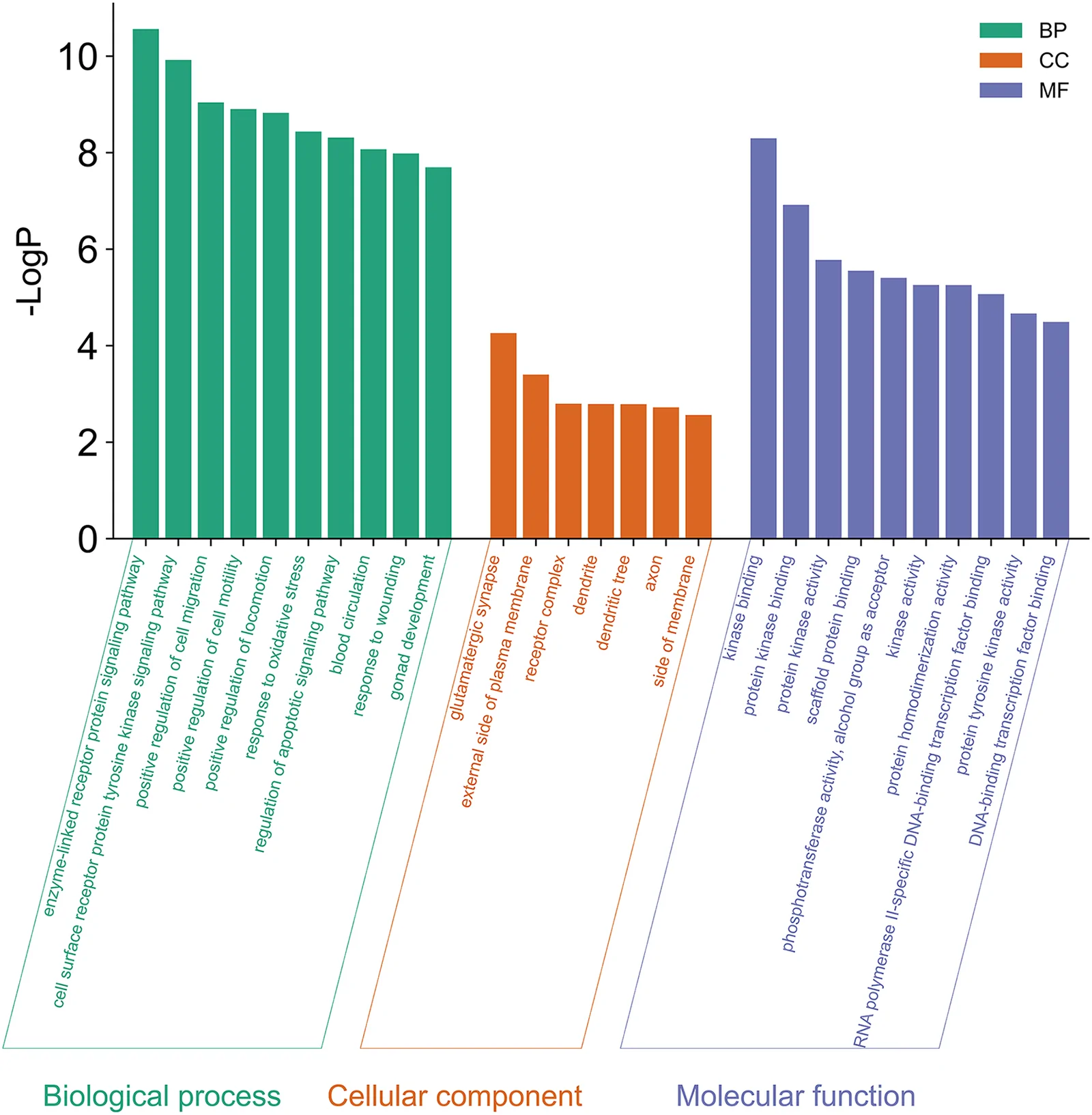
GO enrichment analysis. Including biological processes, cellular components, and molecular functions.

KEGG enrichment results (Fig 5) show that target genes are significantly enriched in classic signaling pathways associated with environmental stress, such as MAPK and PI3K-Akt. Furthermore, the enrichment of the AGE-RAGE and apoptosis pathways suggests a potential association of 6PPD-Q with oxidative stress and mitochondrial dysfunction; The presence of TNF and IL-17 signaling pathways suggests that 6PPD-Q may be involved in the disruption of matrix homeostasis mediated by inflammatory responses within NP cells. Overall, the enrichment results suggest that the potential association between 6PPD-Q and IVDD may involve multiple interconnected pathways related to oxidative stress, inflammation, and apoptosis (S5 Table). To further illustrate the cross-talk relationships between the identified hub genes and enriched signaling pathways, a target–pathway interaction network was constructed using Cytoscape (Fig 6). The network showed that core targets such as PTGS2, MMP2, RELA, SMAD3, and GSK3B were broadly distributed across multiple key pathways, including AGE-RAGE, PI3K-Akt, MAPK, TNF, and IL-17 signaling pathways. Among these, PTGS2, MMP2, and RELA exhibited multiple pathway connections, suggesting that they may serve as important bridging nodes linking oxidative stress, inflammation, apoptosis, and matrix degradation processes in IVDD. These results further support the systematic and multi-target toxicological effects of 6PPD-Q in the progression of IVDD.

**Fig 5:**
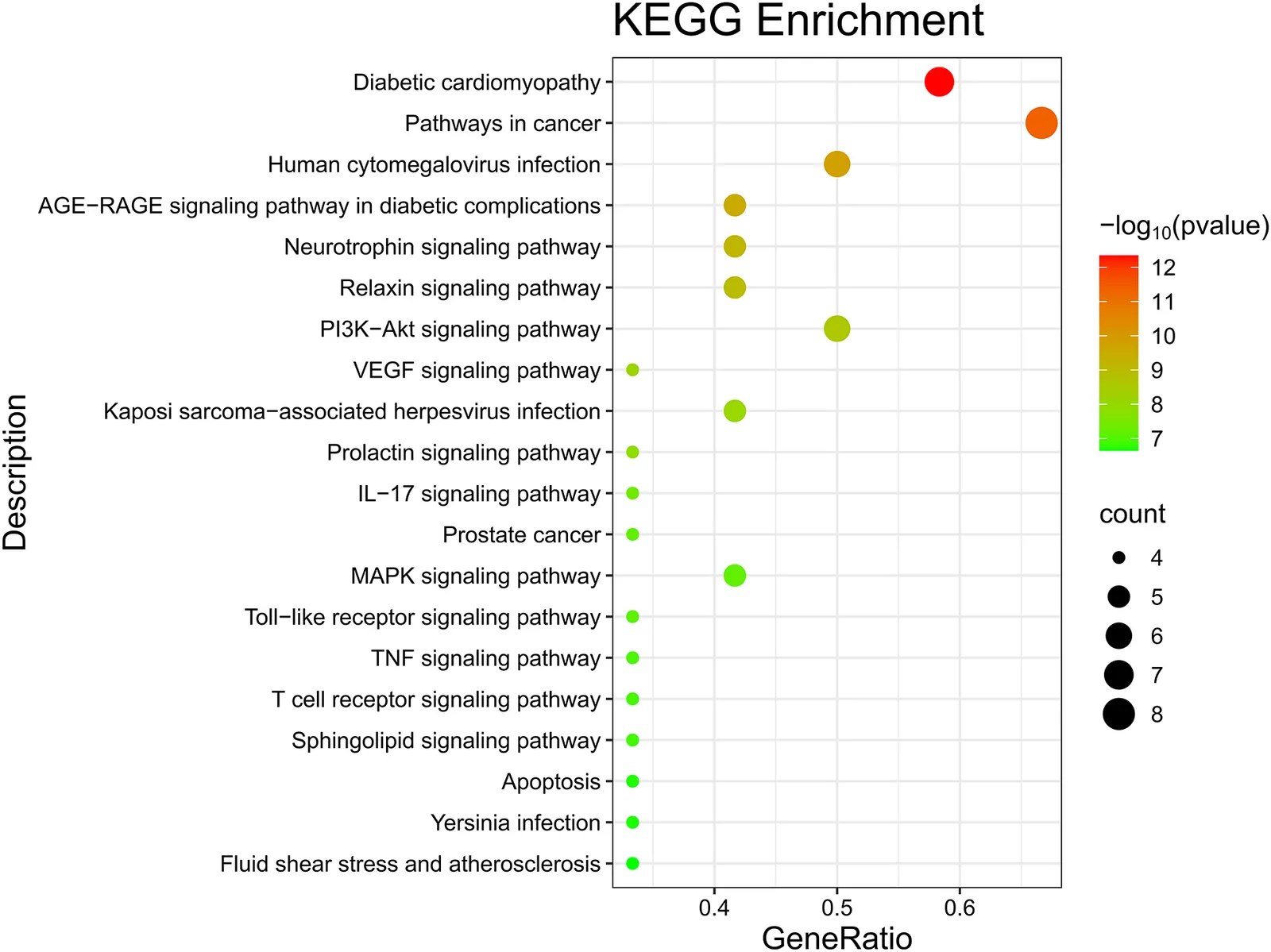
The top 20 KEGG enrichment analysis pathways.

**Fig 6:**
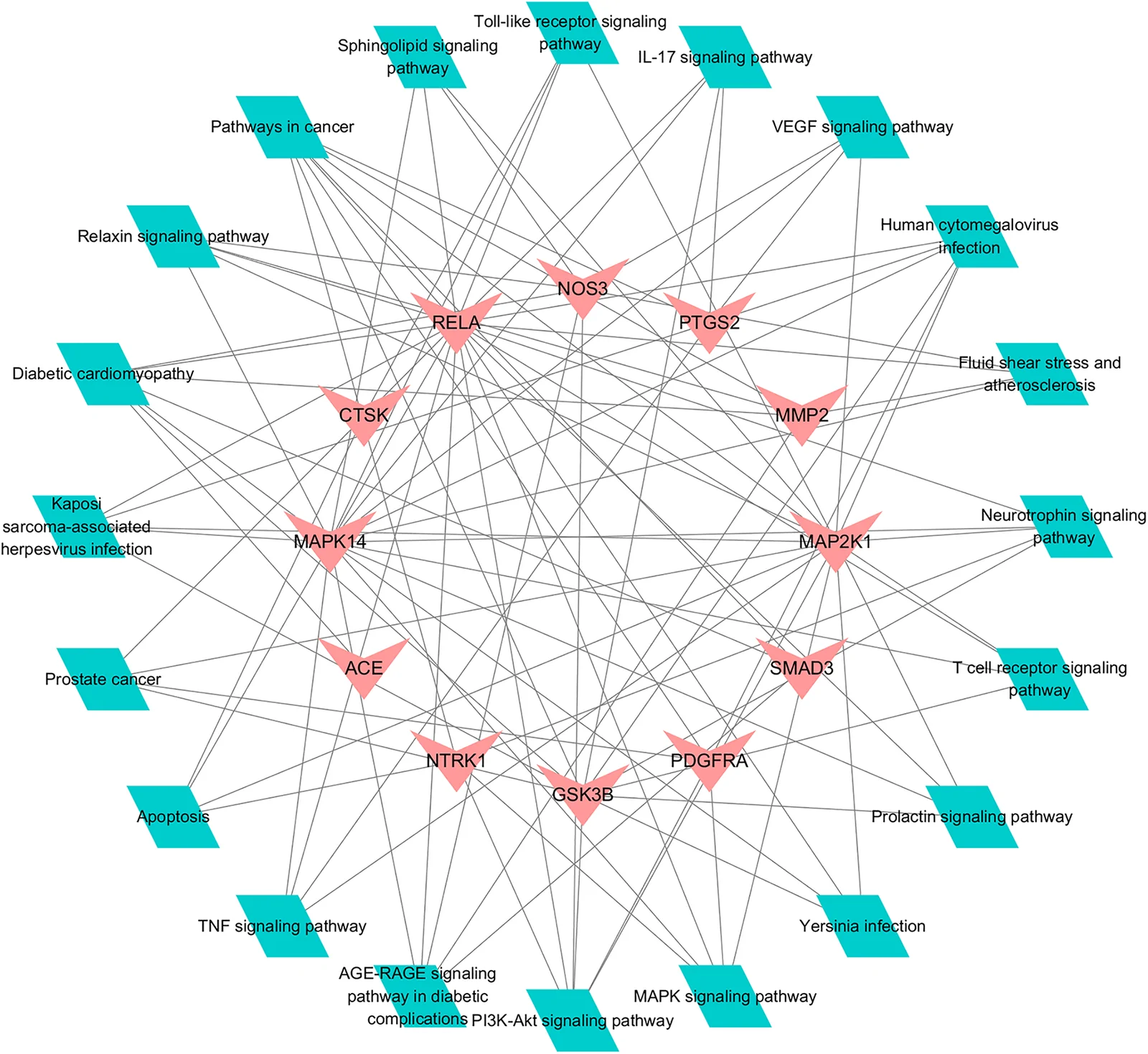
Target–pathway interaction network of core genes and enriched KEGG pathways.

Blue nodes represent core target genes, and red nodes represent signaling pathways. Edges indicate the associations between targets and pathways.

### Molecular docking analysis

To validate the binding affinity between the core targets identified through the PPI network screening and 6PPD-Q at the atomic level, we selected the top five hub genes (PTGS2, MMP2, SMAD3, RELA, GSK3B) for molecular docking. Generally, a binding energy <−5 kcal/mol indicates good binding affinity [36]. The heatmap (Fig 7A) and detailed parameter table (Table 3) show that 6PPD-Q exhibits good binding affinity with all core targets, with binding energies <−6.0 kcal/mol, indicating that thermodynamically stable complexes can be formed between them. Notably, the 6PPD-Q-RELA complex (−8.82 kcal/mol) and the 6PPD-Q-MMP2 complex (−8.30 kcal/mol) exhibited the highest binding stability. Visual analysis further revealed their binding modes (Fig 7B–F): 6PPD-Q can precisely fit into the active pockets of each target protein and maintain conformational stability through hydrogen bonding and hydrophobic interactions. For example, 6PPD-Q forms critical hydrogen bonds with HIS-39 and CYS-47 residues; and establishes a stable bond with the VAL-135 residue of GSK3B. The docking results suggest potential structural interactions between 6PPD-Q and core proteins such as RELA, GSK3B, and MMP2, providing computational support for their possible involvement in inflammation, mitochondrial dysfunction, and matrix degradation.

**Table 3:** Binding parameters between the core target and 6PPD-Q.

Target name	PDB ID	Grid box (X, Y, Z)	Binding energy (kcal/mol)
PTGS2	5f19	(22.594, 40.999, 39.56)	−7.2
MMP2	8H78	(12.277, 19.983, 5.055)	−8.3
SMAD3	1MK2	(9.652, 48.296, 70.154)	−6.09
RELA	8ONV	(16.552, 7.816, 14.574)	−8.82
GSK3B	7B6F	(−0.839, 5.006, 22.148)	−7.5

**Fig 7:**
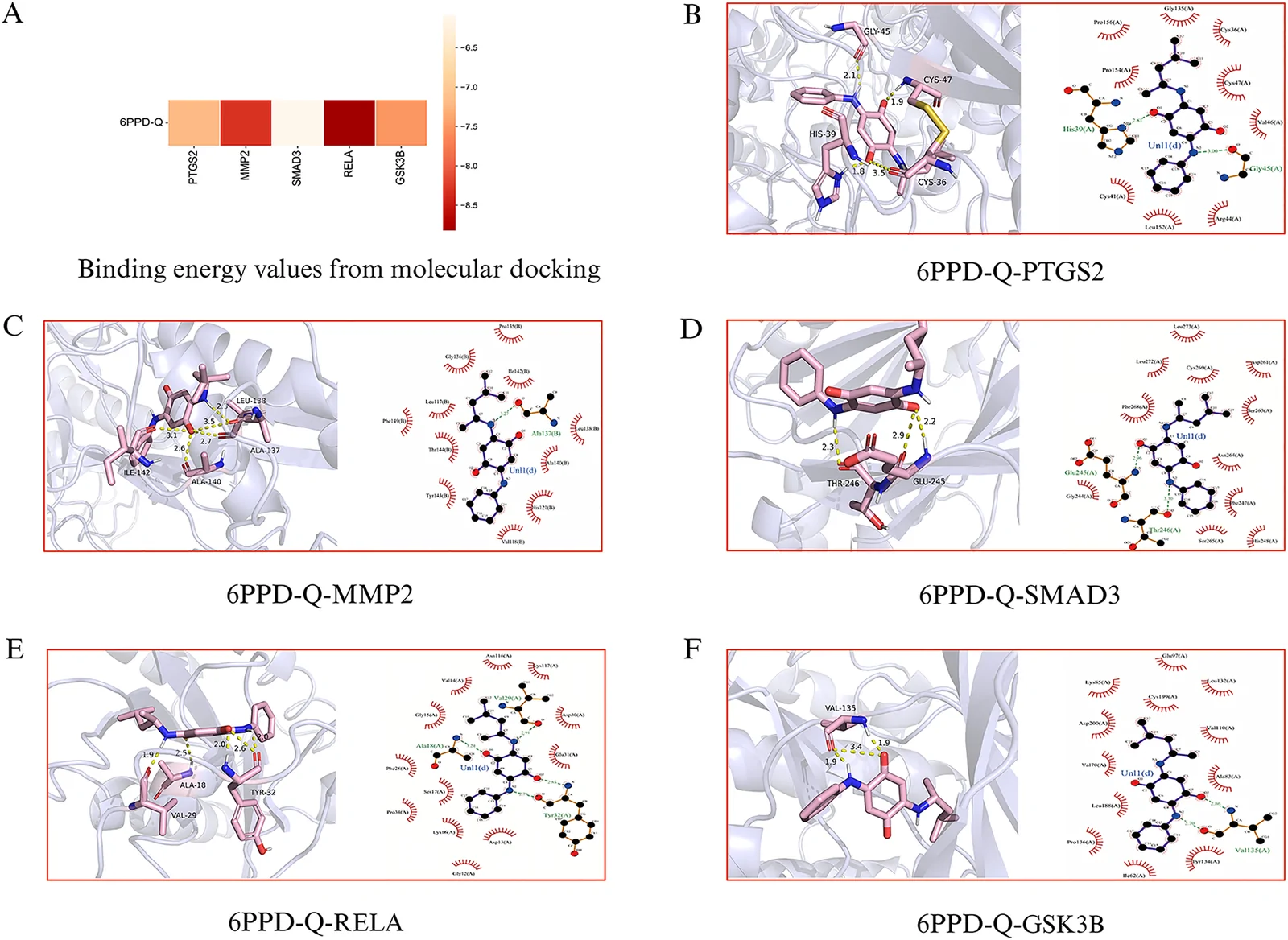
The heat map and molecular docking results of 6PPD-Q biological compounds and core targets. (A) Binding energy value of molecular docking. (B) 6PPD-Q -PTGS2 complex. (C) 6PPD-Q -MMP2 complex. (D) 6PPD-Q -SMAD3 complex. (E) 6PPD-Q -RELA complex. (F) 6PPD-Q -GSK3B complex.

### MD simulation analysis

To further evaluate the dynamic behavior of the optimal docking complex, a 100-ns molecular dynamics simulation was performed for the 6PPD-Q–RELA complex (S6 Table). As shown in Fig 8A, the RMSD profiles of the protein and the protein–ligand complex increased during the initial approximately 15–20 ns and subsequently fluctuated within a relatively narrow range, generally around 0.28–0.35 nm, indicating that the system underwent an initial conformational adjustment followed by a comparatively stable dynamic state. The ligand RMSD remained lower overall, mainly fluctuating between approximately 0.10 and 0.20 nm, although transient increases were observed during the middle stage of the simulation. RMSF analysis showed that most residues exhibited relatively limited fluctuations, generally below 0.20 nm, whereas several localized regions displayed higher flexibility, with peak fluctuations approaching 0.4–0.5 nm (Fig 8B). The solvent-accessible surface area (SASA) remained broadly stable throughout the simulation, varying mainly within approximately 90–98 nm² without persistent abrupt changes, suggesting that no pronounced global unfolding occurred (Fig 8C). Consistently, the overall radius of gyration (Rg) remained highly stable at approximately 1.49–1.53 nm, indicating preservation of the compactness of the protein–ligand complex (Fig 8D). Hydrogen-bond analysis showed that 6PPD-Q maintained intermittent hydrogen-bond interactions with RELA throughout the trajectory, with the number of hydrogen bonds predominantly ranging from 0 to 1 and occasionally reaching 2–3 (Fig 8E). Finally, the free-energy landscape revealed a dominant low-energy basin concentrated at an RMSD of approximately 0.30–0.35 nm and an Rg of approximately 1.51–1.52 nm, suggesting that the complex preferentially sampled a relatively well-defined low-energy conformational state (Fig 8F). Collectively, these results support the dynamic stability of the 6PPD-Q–RELA complex under the simulated conditions.

**Fig 8:**
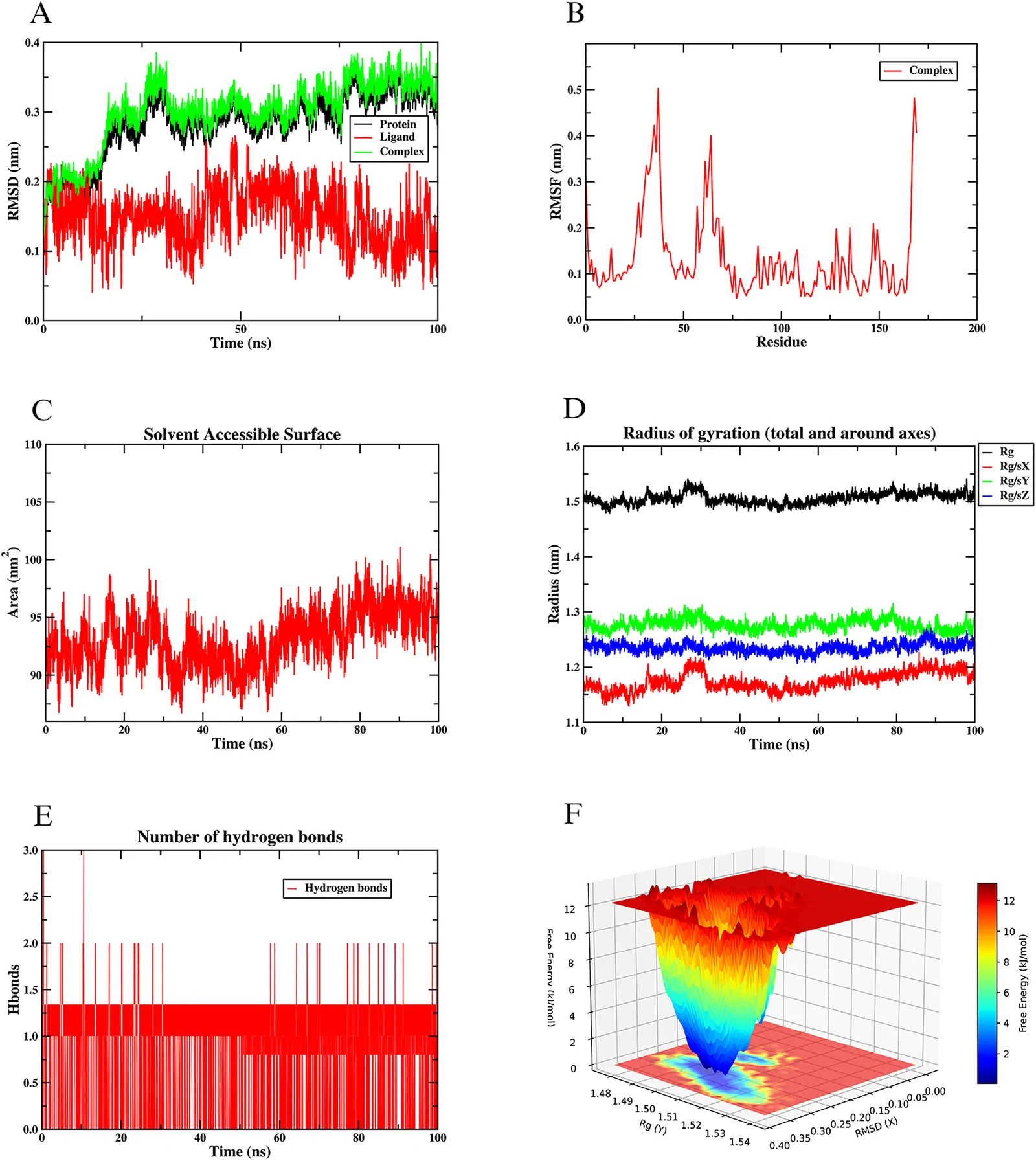
Molecular dynamics simulation analysis of the 6PPD-Q–RELA complex. (A) Root-mean-square deviation (RMSD) profiles of the protein, ligand, and protein–ligand complex during the 100-ns simulation. (B) Root-mean-square fluctuation (RMSF) of protein residues. (C) Solvent-accessible surface area (SASA) of the complex over the simulation trajectory. (D) Radius of gyration (Rg) and its components along the principal axes. (E) Number of hydrogen bonds formed between 6PPD-Q and RELA during the simulation. (F) Free-energy landscape of the 6PPD-Q–RELA complex constructed based on RMSD and Rg.

## Discussion

IVDD is a key pathological basis underlying LBP and the increasing global public health burden [37]. In recent years, 6PPD-Q—a metabolite of tire rubber antioxidants—has emerged as a focus of environmental toxicology due to its widespread exposure in various environmental media and human biological samples [38]. Although preliminary studies have revealed the cellular metabolic toxicity of 6PPD-Q, its potential effects on the avascular skeletal system—particularly on IVD tissue, which has a highly sealed microenvironment—remain unknown. This study explored potential molecular mechanisms linking 6PPD-Q exposure to alterations in IVD microenvironmental homeostasis using a computational toxicology strategy.

Preliminary results from the ADMET-based toxicological prediction platform support our hypothesis. 6PPD-Q not only exhibits potential genotoxicity but also shows a very high propensity for activation in the SR-MMP and SR-ARE models (scores > 0.9 in both cases). In the pathophysiological process of IVDD, the activation of antioxidant response elements (AREs) serves as a marker of cellular response to severe OS, while matrix metalloproteinases (MMPs) are the key drivers of ECM degradation. Given the unique avascular characteristics of adult IVDs, whether and to what extent 6PPD-Q reaches or accumulates in disc tissue remains unclear. Nevertheless, its predicted redox- and matrix-related effects warrant further investigation in the context of IVDD.

Through network toxicology analysis, PTGS2, MMP2, SMAD3, RELA, and GSK3B were identified as hub genes potentially linking 6PPD-Q exposure to IVDD-related processes. Functionally, these targets are highly concentrated in the regulation of inflammatory cascades, matrix metabolism, and mitochondrial homeostasis.

First, PTGS2 (COX-2) and RELA (NF-κB p65) play a key synergistic role in shaping the inflammatory microenvironment. Under pathological stimulation, the phosphorylation and nuclear translocation of RELA can broadly upregulate the expression of pro-inflammatory factors [39,40]; simultaneously, PTGS2, as the rate-limiting enzyme in the inflammatory cascade, often exhibits compensatory upregulation in response to exogenous chemical toxicity [41], thereby exacerbating local inflammatory damage and discogenic pain [42]. As a quinone pollutant with intrinsic redox activity, the accumulation of 6PPD-Q within the NP can directly induce the production of reactive oxygen species (ROS) [43]. This exogenous OS acts as an upstream signal, strongly activating the NF-κB pathway, amplifying the p65-mediated inflammatory cascade, and leading to chronic inflammatory imbalance in IVDD.

Second, the progressive loss of the ECM is a key hallmark of structural degeneration in IVDD. This process is governed by a dynamic interplay between matrix degradation (mediated by MMP2) and anabolic processes (mediated by SMAD3) [44,45]. As an enzyme that specifically cleaves type II collagen and aggregated proteoglycans, excessive MMP2 activity severely compromises the physical load-bearing capacity of the cartilage and NP microenvironment [46]. Genetic evidence also suggests that specific MMP2 polymorphisms (such as the −1306CC genotype) significantly increase the risk of IVDD in the general population [47]. Notably, activation of the AGE-RAGE signaling pathway may lead to IVDD by accelerating MMP2 expression [48]. Conversely, SMAD3, as a key transcription factor in the TGF-β pathway, is responsible for maintaining the synthesis of endogenous matrix macromolecules [49,50]. Therefore, if the phosphorylation of SMAD3 or upstream signaling is inhibited, the endogenous matrix repair capacity of NP cells will significantly decline, further exacerbating the progressive loss of the ECM and the pathological progression of IVDD [51,52]. The network analysis in this study is highly consistent with ADMET predictions, suggesting that exposure to 6PPD-Q may inhibit SMAD3-mediated matrix repair capacity while indirectly upregulating MMP2 expression via OS, thereby accelerating ECM depletion.

Furthermore, the key to maintaining the viability of NP cells lies in mitochondrial homeostasis, and GSK3B is the central kinase regulating this process [53]. During the progression of IVDD, hyperactive GSK3B promotes the opening of the mitochondrial permeabilization transition pore (mPTP), accelerating the release of cytochrome C; simultaneously, it inhibits PINK1/Parkin-dependent mitochondrial autophagy, leading to the accumulation of damaged mitochondria and the sustained production of reactive oxygen species (ROS) [54]. Existing toxicological evidence has confirmed that 6PPD-Q possesses direct mitochondrial toxicity and can significantly disrupt cellular energy metabolism [55]. It is therefore hypothesized that 6PPD-Q may target GSK3B, exacerbating the collapse of energy metabolism and programmed apoptosis in NP cells.

Enrichment analysis further suggested the systemic and complex nature of the potential toxicological effects of 6PPD-Q. Target genes were significantly enriched in pathways such as AGE-RAGE, MAPK, PI3K-Akt, and TNF/IL-17, delineating a clear toxic pathway involving “stress perception–signal transduction–effect execution.” Specifically, as a pro-oxidant, 6PPD-Q may affect upstream AGE-RAGE and MAPK stress-related signaling, potentially through ROS-associated mechanisms [56,57]. The abnormal activation of these pathways not only inhibits classical cellular survival pathways such as PI3K-Akt [58,59] but also acts as a cascade amplifier, triggering downstream TNF- and IL-17-mediated inflammatory responses [60]. These findings suggest that 6PPD-Q exposure may represent a potential environmental factor associated with this pathological cascade. Its potential diffusion and accumulation within the avascular IVD may be associated with alterations in oxidative stress-related and inflammatory signaling, thereby potentially contributing to the inflammatory microenvironment of IVDD [61]. The target–pathway interaction network further showed that hub genes such as PTGS2, MMP2, and RELA were involved in multiple enriched pathways, supporting a coordinated multi-target and multi-pathway mechanism through which 6PPD-Q may contribute to IVDD rather than acting through a single isolated signaling cascade.

In summary, the aforementioned network analysis identified several key pathways potentially linking 6PPD-Q exposure to disruption of IVD microenvironmental homeostasis. However, the molecular interactions between 6PPD-Q and the identified hub proteins require further structural and experimental evaluation. Molecular docking suggested favorable predicted binding between 6PPD-Q and all five hub proteins, with the 6PPD-Q–RELA (−8.82 kcal/mol) and 6PPD-Q–MMP2 (−8.30 kcal/mol) complexes showing the most favorable docking scores. Given the static nature of molecular docking, the 6PPD-Q–RELA complex was further subjected to a 100-ns molecular dynamics simulation. The relatively stable RMSD, Rg, and SASA profiles, together with the concentrated low-energy conformational basin observed in the free-energy landscape, provided additional computational support for the dynamic stability of the predicted 6PPD-Q–RELA complex under the simulated conditions. These findings suggest a potential structural basis for the interaction of 6PPD-Q with inflammation-related targets such as RELA; however, direct molecular binding and downstream biological effects remain to be experimentally validated.

Finally, this study has several limitations. First, the current findings are primarily based on database-derived computational analyses, molecular docking, and molecular dynamics simulations. Although the MD simulation provides additional information regarding the dynamic behavior and conformational stability of the predicted 6PPD-Q–RELA complex under the simulated conditions, these computational approaches cannot establish direct molecular binding or causal biological effects. Therefore, the predicted targets, interactions, and signaling pathways require further validation using NP cell models, biochemical binding assays, and in vivo experiments. Second, in real-world settings, humans may be exposed to mixtures of 6PPD, 6PPD-Q, and other related transformation products; focusing on a single compound may therefore underestimate the complexity of cumulative exposure and toxicity. Furthermore, general-purpose databases and computational models cannot fully recapitulate the unique avascular, hypoxic, and mechanically stressed microenvironment of the intervertebral disc. Future studies should therefore further investigate the tissue-specific pharmacokinetics, exposure characteristics, and biological effects of 6PPD-Q in the IVD.

## Conclusion

By integrating network toxicology, molecular docking, and molecular dynamics simulation, this study explored the potential molecular links between 6PPD-Q exposure and IVDD. The results suggest that oxidative stress, mitochondrial dysfunction, and inflammatory signaling may be involved, with AGE-RAGE, PI3K-Akt, MAPK, TNF, and IL-17 signaling pathways emerging as potentially relevant networks. Five hub genes, PTGS2, MMP2, SMAD3, RELA, and GSK3B, were identified. Molecular docking suggested potential interactions between 6PPD-Q and these hub proteins, while subsequent 100-ns MD simulation provided additional computational support for the dynamic stability of the 6PPD-Q–RELA complex under the simulated conditions. These findings provide a computational framework and testable hypotheses for understanding the potential association between 6PPD-Q exposure and IVDD. Further in vitro and in vivo studies are required to validate the predicted molecular interactions and biological mechanisms.

## Supporting information

S1 Table PubChem information for 6PPD-Q. (XLSX)

S2 Table Potential targets of 6PPD-Q retrieved from different target prediction databases. (XLSX)

S3 Table IVDD-associated genes retrieved from different disease-related databases. (XLSX)

S4 Table Common targets between 6PPD-Q and intervertebral disc degeneration. (XLSX)

S5 Table Detailed results of GO and KEGG enrichment analyses. (XLSX)

S6 Table Underlying numerical data for molecular dynamics simulation analyses of the 6PPD-Q–RELA complex. (XLSX)
